# Supplementary material for: The freeze-avoiding mountain pine beetle (Dendroctonus ponderosae) survives prolonged exposure to stressful cold by mitigating ionoregulatory collapse
Source: J Exp Biol. 2024 May 7;227(9):jeb247498. doi: 10.1242/jeb.247498 (PMC11128280; doi:10.1242/jeb.247498)
Supplement: Supplementary information [file jexbio-227-247498-s1.pdf]

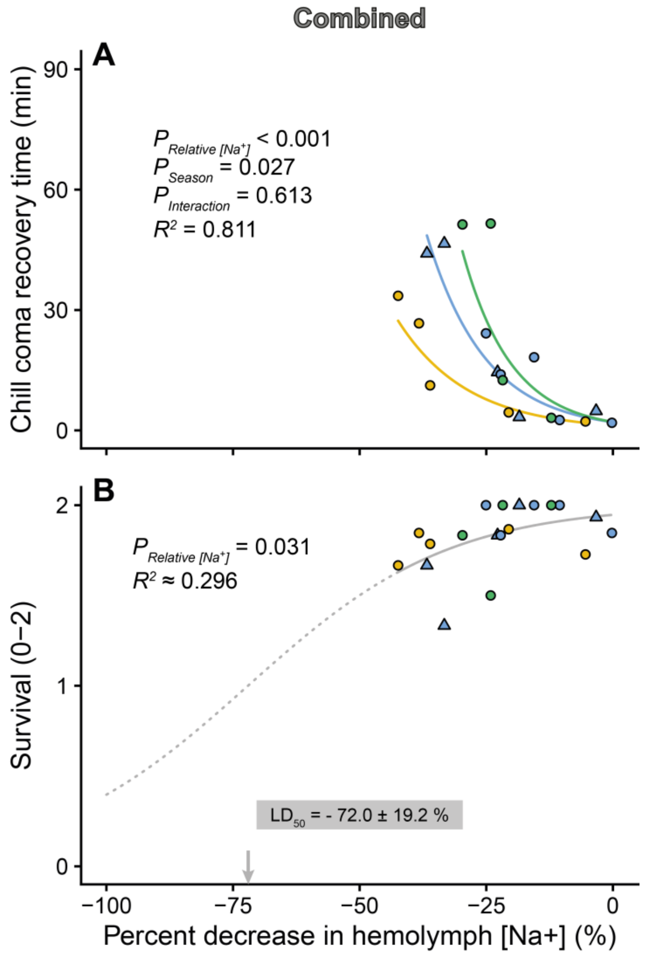

Fig. S1. Correlations between the degree of hemolymph hyponatremia and cold tolerance phenotypes. These correlations were made using measurements of CCRT, survival score, and hemolymph Na^+ concentration obtained at the same time points from autumn (yellow), winter (blue), and spring (green), and at both -10°C (circles) and -20°C (which was only measured in winter). Note that these measurements are from a subsample of time points. Overall there was a strong exponential relationship between the degree of hemolymph hypernatremia and the CCRT (**A**), and this relationship was season-specific such that recovery times were shortest in the fall, followed by winter and then spring (no effect of exposure temperature were found). In terms of the survival score (B), there were no effects of temperature or seasonality and survival was found to decrease as a function hyponatremia. Like with hemolymph hyperkalemia, we fitted a sigmoidal model to the data which we used to extrapolate and find a LD50 at a -72.0 ± 19.2% decrease in hemolymph Na^+ concentration.

Journal of Experimental Biology • Supplementary information

Table S1. This table contains the names and coordinates of the three weather stations used to approximate the thermal conditions of the threes containing *D. ponderosae* larvae before being transported to University of Alberta. Data was downloaded on October 18, 2023, and covered the period from September 1, 2022, to May 31, 2023.

Weather station name	Latitude	Longitude
Rocky Mtn House A	52,4333	-114,9167
Edson	53,5833	-116,4167
Edmonton Stony Plain CS	53,5472	-114,1084

Table S2. Sample sizes for data presented in Fig. 2, panels B-D, where supercooling points were measured at three different time points in larvae of *D. ponderosae* during overwintering.

**Season**	Supercooling point **Sample size (N)**
Autumn	19
Winter	22
Spring	12

Table S3. Sample sizes for data presented in **Fig. 3**, where chill coma recovery time and survival score were quantified as a function of seasonal time point, exposure time, and exposure temperature. Note that even though the chill coma recovery time and survival score were quantified in the same animals (see Materials and Methods), the sample size for survival score is often higher than that of chill coma recovery time. This is because animals that did not recover in the 90 min time limit were counted as having not recovered and were therefore excluded from any analysis related to recovery times.

**Season**	**Exposure temperature (°C)**	**Exposure time (h)**	Chill coma recovery time **Sample size (N)**	Survival score **Sample size (N)**
Autumn	-10	0	11	11
Autumn	-10	4	14	15
Autumn	-10	16	18	18
Autumn	-10	24	14	14
Autumn	-10	48	12	13
Autumn	-10	96	11	12
Autumn	-10	168	12	13
Winter	-20	0	15	15
Winter	-20	4	13	13
Winter	-20	16	12	14
Winter	-20	24	10	12
Winter	-20	48	15	16
Winter	-20	96	6	12
Winter	-20	168	9	12
Winter	-10	0	12	13
Winter	-10	4	16	16
Winter	-10	16	15	15
Winter	-10	24	16	16
Winter	-10	48	17	17
Winter	-10	96	11	12
Winter	-10	168	12	12
Spring	-10	0	13	13
Spring	-10	4	12	12
Spring	-10	16	16	16
Spring	-10	24	13	13
Spring	-10	48	11	11
Spring	-10	96	11	16
Spring	-10	168	18	18

Journal of Experimental Biology • Supplementary information

Table S4. Sample sizes for data presented in Fig. 4, where hemolymph concentrations of K^+ and Na^+ were measured in overwintering larvae of *D. ponderosae* exposed to stressful low temperature for different amounts of time. Note that the sample size for Na^+ concentration is lower than that for K^+ concentration at the spring season for animals exposed to -10°C for 168 h because some samples were lost.

Season	Exposure temperature (°C)	Exposure time (h)	Hemolymph K^+ concentration **Sample size (N)**	Hemolymph Na^+ concentration **Sample size (N)**
Autumn	22	Room temperature control	12	-
Spring	22	Room temperature control	15	-
Winter	22	Room temperature control	12	-
Autumn	6	6°C control	9	9
Spring	6	6°C control	15	15
Winter	6	6°C control	7	7
Winter	-20	0	12	12
Autumn	-10	0	12	12
Spring	-10	0	16	16
Winter	-10	0	10	10
Winter	-20	4	13	13
Autumn	-10	4	14	14
Spring	-10	4	10	10
Winter	-10	4	12	12
Winter	-20	16	15	-
Autumn	-10	16	15	-
Spring	-10	16	16	-
Winter	-10	16	12	-
Winter	-20	24	10	10
Autumn	-10	24	13	13
Spring	-10	24	15	15
Winter	-10	24	9	9
Winter	-20	48	16	-
Autumn	-10	48	11	-
Spring	-10	48	9	-
Winter	-10	48	17	-
Winter	-20	96	6	6
Autumn	-10	96	12	12
Spring	-10	96	16	16
Winter	-10	96	7	7
Winter	-20	168	9	9
Autumn	-10	168	11	11
Spring	-10	168	17	12
Winter	-10	168	8	8

Dataset 1.

Available for download at
https://journals.biologists.com/jeb/article-lookup/doi/10.1242/jeb.247498#supplementary-data

Journal of Experimental Biology • Supplementary information
